# 

**Published:** 1954-07

**Authors:** 


MEDICAL JOURNAL OF THE SOUTH-WEST

*(Incorporating the Bristol Medico-Chirurgical Journal)*

Established 1883

VOL. 70 (iii) July, 1954 NO. 255

### Contents

*PUBLISHED QUARTERLY*

*ANNUAL SUBSCRIPTION 16/- POST FREE*

PUBLISHED UNDER THE AUSPICES OF THE
BRISTOL MEDICO-CHIRURGICAL SOCIETY

### Editorial Committee

Mr. H. Keith Lucas, 60 St. John's Road, Bristol 8. *Editor.*

Dr. J. E. Cates, Dept. of Medicine, Bristol Royal Infirmary } *Assistant Editors.*
Dr. A. H. Gale, The University, Bristol 8 }

Dr. J. Macrae, Ham Green Hospital. *Treasurer.*

Mr. J. P. Partridge, Dept. of Surgery, Bristol Royal Infirmary.

### *Local Correspondents*

*Bath* . . Mr. A. D. Bateman, 3 The Circus, Bath.
*Exeter* . . Dr. L. N. Jackson, Mount Jocelyn, Crediton, Devon.
*Gloucester* . Dr. R. F. Jarrett, 9 College Green, Gloucester.
*Plymouth* . Dr. C. R. Croft, Lexden, Hartley Av., Plymouth.
*Taunton* . Dr. M. McAlley, 1 The Crescent, Taunton.
*Torquay* . Dr. A. Robinson Thomas, 20 Devon Square, Newton Abbot, Devon.
*Truro* . . Dr. F. D. M. Hocking, St. Andrews, Perranporth, Cornwall.
*Yeovil* . . Mr. J. A. Vere Nicoll, Knapp House, Yeovil, Somerset.

### NOTICE TO CONTRIBUTORS

Original articles are invited, provided they have not been published elsewhere. Notes of forthcoming events, meetings held, degrees conferred, staff appointments, and other local news are welcomed. The editorial committee reserves the right to make literary corrections.

Illustrations should always be supplied ready for reproduction. All re-touchings or other operations required will be charged to the author. Line drawings should be drawn in Indian ink. We regret that we must ask authors to pay for making blocks, if this is necessary.

J. W. ARROWSMITH LTD., SMALL STREET,
BRISTOL.

### Notes and News

Mr. W. A. Jackman, F.R.C.S. (Ed.), has been elected to the Fellowship of the Royal College of Surgeons of England *ad eundem*.

Dr. J. M. Naish has been elected to the Fellowship of the Royal College of Physicians of London.

Mr. J. H. Peacock, F.R.C.S., has been awarded the Jacksonian Prize of the Royal College of Surgeons of England.

Dr. J. Creighton Bramwell, of Manchester, will deliver the Carey Coombs Memorial Lecture in 1954 on " Rheumatic Valvular Disease 1924–54 ".

Mr. Norman Tanner, F.R.C.S., will deliver the Long Fox Memorial Lecture on a subject to be announced later.

Professor Bruce T. Mayes, Professor of Obstetrics in Sydney, gave a lecture at Southmead on May 16th on " Post-partum Haemorrhage and the Management of the Third Stage of Labour ".

A week-end course for general practitioners was held at Exeter in the week-end May 14th–17th. A week's course will be held in Bristol from July 11th to 17th. Next session the usual series of Sunday morning demonstrations will probably be arranged in Bristol.

Messrs. John Wright & Sons have inaugurated a travelling book showroom for the sale of medical books to the staffs of peripheral hospitals in the South West.

### EXAMINATION RESULTS

*M.R.C.P. (London):* M. D. Turner.
*F.R.C.S. (Ed.):* E. R. Duchesne.
*D.A.:* C. D. Linton.
*D.P.M. (Bristol):* L. W. Bowen, D. E. Jarman, S. Prus.
*D.C.H.:* Miss A. M. Haines, Miss H. Patel, Miss F. E. Powell, Mrs. R. M. Walters, G. R. Wijegunaratne.

### PRIZES

*Francis Dudley Memorial Prize for* 1953: Awarded jointly to Miss D. O'Connor and Mr. M. G. Addy.

*Dental Gold Medal:* Awarded to Mr. J. E. Bowerman.

*Hewer Pathology Prize for* 1954: Awarded to Mr. G. A. J. Ayliffe and Mr. K. R. Gough.

*Foyle Prize for* 1954: Awarded to Mr. R. T. Marcus.

### *Appointments*

### *UNITED BRISTOL HOSPITALS*

| *Name* | *Specialty* | *Where from* | *Commenced* |
|---|---|---|---|
| MACGILLIVRAY, IAN, M.B., CH.B., M.R.C.O.G., M.D. (*Hon. Appointment*) | Obstetrics and Gynaecology. | Was previously lecturer in Dept. of Midwifery, University of Glasgow, and Clinical Assistant, Glasgow Royal Maternity and Women's Hospital and Western Infirmary, Glasgow. | 1.1.54 |
| PRICE, ARTHUR KENNETH, M.B., B.S., F.R.C.S., M.R.C.S., L.R.C.P. (*Hon. Appointment*) | Thoracic Surgery. | Consultant in Thoracic Surgery, South Western Regional Hospital Board (Frenchay Hospital). | 1.1.54 |

| *Name* | *Previous Post* | *Post appointed to* | *Commencing date* |
|---|---|---|---|
| CAMPBELL, MISS D. M. Y., L.D.S. | Senior Orthodontic Registrar, King's College Hospital Dental School. | Senior Registrar in Orthodontics. | Jan., 1954 |
| LEATHER, H. M., M.B., CH.B., M.R.C.P. | Medical Registrar, Manchester Royal Infirmary, and Resident Medical Officer, Barnes Hospital, Cheadle. | Senior Medical Registrar. | Mar., 1954 |
| HILL, R., M.B., CH.B., M.R.C.P. | Medical S.H.O., Southmead Hospital, Bristol. | Medical Registrar. | Mar., 1954 |
| JOHNSON, D. H., M.B., CH.B., M.D. | Registrar in Pathology, United Bristol Hospitals. | Senior Registrar in Pathology. | Mar., 1954 |
| MOORE, G. E., L.D.S., H.D.D., F.D.S. | Dental House Surgeon, Worcestershire, Mid-District. | Registrar in Dental Surgery. | Mar., 1954 |
| MONKS, P. J. W., M.B., B.S., F.R.C.S. | Surgical Registrar, Southmead Hospital, Bristol. | Senior Surgical Registrar. | Apl., 1954 |
| CROW, R. S., M.B., CH.B., M.R.C.P. | Senior Medical Registrar, Kingston Hospital, Surrey. | Senior Medical Registrar. | Apl., 1954 |
| FORREST, MISS G. M. S., M.B., CH.B. | Senior House Officer Anaesthetist, Cheltenham General, Eye and Children's Hospital. | Registrar in Anaesthetics. | Apl., 1954 |
| NOBLE, D. S., M.B., B.S., B.D.S., L.D.S., R.C.S. | House Surgeon, Guy's Hospital Dental School. | Registrar in Dental Surgery. | Apl., 1954 |
| SPENCER, R. W., M.B., CH.B., M.R.C.P. | Junior Assistant Pathologist, John Bonnett Laboratory, Cambridge University. | Senior Registrar in Pathology. | May, 1954 |
| MOORE, E. W., M.B., CH.B., D.P.H., M.R.C.P. | Part-time Registrar in Diagnostic Radiology, United Bristol Hospitals. | Registrar in Radiology (Diagnostic). | June, 1954 |
| HARDY, H. M., M.B., CH.B. | Registrar in Anaesthetics, United Sheffield Hospitals. | Registrar in Anaesthetics. | June, 1954 |

### *BRISTOL*

| *Name* | *Appointment* | *Formerly* |
|---|---|---|
| LITTLE, BRIAN R., M.R.C.S., L.R.C.P., D.A.(ENG.), F.F.A.R.C.S. | Consultant Anaesthetist, Bristol Clinical Area (Weston-super-Mare). | Consultant Anaesthetist, North Gloucestershire, Clinical Area. |
| SLEIGH, BEATRICE E., M.B., CH.B., D.A. | Anaesthetic Registrar, Frenchay Hospital, Bristol. | Anaesthetic Registrar, United Bristol Hospitals. |
| TULLOCH, L. G., M.B., CH.B. (ABERDEEN), M.D. (ABERDEEN). | Senior Registrar in Dermatology (Joint appointment with the United Bristol Hospitals). | Registrar in Dermatology, Manchester and Salford Hospital for Diseases of the Skin. |
| HOUGHTON, E. A. W., M.B., CH.B. (BRISTOL). | Medical Registrar, Southmead Hospital, Bristol. | S.H.O. in General Medicine, Southmead Hospital. |
| KNOWLES, R. R., M.B., B.S. (SYDNEY), D.P.M. | Senior Registrar in Psychiatry, Bristol Mental Hospitals. | Psychiatric Registrar, Blackburn Royal Infirmary. |

### *NORTH GLOUCESTERSHIRE*

| *Name* | *Appointment* | *Formerly* |
|---|---|---|
| REES, DAIREEN I., M.R.C.S., L.R.C.P. | General Practitioner Anaesthetist, Stroud General Hospital (2 sessions). | Is in general practice at Nailsworth. |
| ROWLANDS, IRENE P., M.R.C.S., L.R.C.P., F.R.F.P.S., M.R.C.P. (EDIN. & LONDON). | S.H.M.O. Geriatrician, North Gloucestershire Clinical Area. | Medical Registrar, Hereford Group of Hospitals. |

### *BATH*

| | | |
|---|---|---|
| BURROWES, W. L. M.B., B.CH. (BELFAST), M.D., M.R.C.P.I. | Clinical Assistant in General Medicine, Royal United Hospital, Bath (1 session). | Is in general practice at Corsham. |

### *EXETER*

| *Name* | *Appointment* | *Formerly* |
|---|---|---|
| TROBRIDGE, G. F., M.B., CH.B. (B'HAM). | Registrar in Diseases of the Chest, Hawkmoor Chest Hospital, Bovey Tracey. | Registrar, Leeds Chest Clinic. |
| DORAN, H. J., M.B., B.CH. (BELFAST), D.L.O. | Clinical Asst. in E.N.T. Surgery, Newton Abbot and Dawlish Hospitals (2 sessions). | Is in general practice at Torquay. |

### *DEVON AND CORNWALL*

| *Name* | *Appointment* | *Formerly* |
|---|---|---|
| BRAMLEY, P. A., L.D.S., R.C.S. (ENG.), L.D.S. (B'HAM), M.B., CH.B. (B'HAM), F.D.S., R.C.S. (ENG.). | Consultant Dental Surgeon, Plymouth Clinical Area (4 sessions). | Dental Registrar, Rooksdown House, Plastic and Jaw Unit, Basingstoke. |
| COLLINS, JOAN R., M.B., CH.B. (B'HAM). | General Practitioner Anaesthetist, Newquay and District Hospital (2 sessions). | Is in general practice at Newquay. |
| TRUSCOTT, D. E., M.B., B.S., D.M.R.(D.) (LOND.), D.M.R.(D.) (CONJOINT BOARD). | Consultant Radiologist, based at West Cornwall Hospital, Penzance. | Senior Registrar in Radiology, Glasgow Royal Infirmary. |

